# An ethnomethodological approach to examine exploitation in the context of capacity, trust and experience of commercial surrogacy in India

**DOI:** 10.1186/1747-5341-8-10

**Published:** 2013-08-20

**Authors:** Sheela Saravanan

**Affiliations:** 1 Department of Medical Ethics and History of Medicine, Institute, University Medical Center, Humboldtallee 36, Goettingen D-37073, Germany

**Keywords:** Commercial surrogacy, Manifestations of exploitation, Capacity, Asymmetric vulnerability, Trust, Human relationships, Bio-power

## Abstract

The socio-ethical concerns regarding exploitation in commercial surrogacy are premised on asymmetric vulnerability and the commercialization of women’s reproductive capacity to suit individualistic motives. In examining the exploitation argument, this article reviews the social contract theory that describes an individual as an ‘economic man’ with moral and/or political motivations to satisfy individual desires. This study considers the critique by feminists, who argue that patriarchal and medical control prevails in the surrogacy contracts. It also explores the exploitative dynamics amongst actors in the light of Baier’s conceptualization of trust and human relationship, within which both justice and exploitation thrive, and Foucault’s concept of bio-power. Drawing on these concepts, this paper aims to investigate the manifestations of exploitation in commercial surrogacy in the context of trust, power and experiences of actors, using a case study of one clinic in India. The actors’ experiences are evaluated at different stages of the surrogacy process: recruitment, medical procedures, living in the surrogate home, bonding with the child and amongst actors, financial dealings, relinquishment and post-relinquishment.

This study applies ethnomethodology to identify phenomena as perceived by the actors in a situation, giving importance to their interpretations of the rules that make collective activity possible. The methods include semi-structured interviews, discussions, participant observation and explanation of the phenomena from the actors’ perspectives. Between August 2009 and April 2010, 13 surrogate mothers (SMs), 4 intended parents (IPs) and 2 medical practitioners (MPs) from one clinic in Western India were interviewed.

This study reveals that asymmetries of capacity amongst the MPs, SMs, IPs and surrogate agents (SAs) lead to a network of trust and designation of powers through rules, bringing out the relevance of Baier’s conceptualization of asymmetric vulnerability, trust and potential exploitation in human relationships. The IPs are exploited, especially in monetary terms. The SMs are relatively the most exploited, given their vulnerability. Their remuneration through surrogacy is significant for them, and their acquired knowledge as ex-surrogates is used for their own benefit and for exploiting others. Foucault’s conceptualization of power is hence relevant, since the ex-SMs re-invest the power of their exploitative experience in exploiting others.

## Introduction

India is one of the popular global health destinations providing medical care, equipment and facilities at a comparatively lower cost [1]. A subset of medical tourism is reproductive health care, including treatments such as assisted reproductive technology (ART) and surrogacy. The extent of this rapidly expanding enterprise in India is unclear, due to the lack of adequate official data. According to official figures, about 3000 registered clinics across India offer surrogacy services [2]. However, the Medical Director of the Reproductive Medicine and Women’s Health Unit at a hospital in Chennai stated that over 30000 ART clinics in the country are fully equipped for supporting surrogacy [3]. Dr. Anita Soni, a physician at Hiranandani Hospital, says she delivers babies of Indian women for British couples at the rate of more than 15 a month [4]. The Government of India legalized surrogacy with the ART Regulation Bill [5]. However, the ART Bill has remained in a draft form since 2010. Several unclear clauses within the draft bill are criticized as discriminatory towards the rights of the surrogate mothers (SMs), especially by feminist groups within India [6,7]. Several reviews on commercial surrogacy in India from the women’s rights [8] and legal perspectives [6,7] are available, and recently there have been empirical narratives from sociological and anthropological standpoints [9,10]. However, empirical research examining the exploitation argument within the process of commercial surrogacy from an ethical perspective is lacking in India. This paper aims to examine the manifestations of exploitation in commercial surrogacy with a case study of one clinic in Western India. From 2003 to 2009, this clinic had successfully delivered 179 babies through 136 surrogacy cases for couples, both from overseas and within India.

## Human relationships in surrogacy contracts

The socio-ethical concerns of exploitation in commercial surrogacy are premised on asymmetric vulnerability and the commercialization of women’s reproductive capacity to suit individualistic motives. In examining the exploitation argument, this article reviews the social contract theory that describes an individual as an ‘economic man’ with moral and/or political motivations to satisfy individual desires. It also evaluates the critique by feminists, who argue that patriarchal and medical control prevails in the surrogacy contracts. This study also examines Baier’s conceptualization of human relationship in terms of trust, within which both justice and exploitation thrive, and the conceptualization of power within the theory of structuration and Foucault’s concepts on bio-power. The social contract theory refers to individuals with moral and/or political obligations who are dependent upon a contract or agreement to form the society in which they amicably live. According to Hobbes, individuals are not only self-interested, but also reasonable beings motivated by individual desires [11]. Hence, they will choose to submit to governmental authority through a social contract to be able to live in a civil society. Contractarianism follows the Kantian understanding of persons and their capacities. Following the Kantian approach, John Rawls describes the contractarian framework, wherein individuals have the capacity to reason from a universal point of view, which means that they have the moral capability of judging principles from an impartial standpoint [12]. In her book, *The Social Contract*, Carole Pateman critiques the social contract theory and asserts that patriarchal control prevails in the marriage contract, the prostitution contract, and the contract for surrogate motherhood [13]. She claims surrogacy contracts are the means by which women’s reproductive capacities are dominated and patriarchy is upheld. In *Feminist Morality*, Virginia Held regards the social contract theory as inadequate in representing children and women and in capturing the meaningful moral relationship between people [14]. Similarly, Annette Baier notes that ‘the liberal individual’ in the social contract theory only defines individual rights and obligations, and does not sufficiently reveal the full extent of a moral individual and the interdependent relations between individuals [15].

Baier notes that vulnerability and asymmetrical dependence that are more common in human relations should be of greater concern to ethicists, rather than structured agreements, as described in the social contract theory. In her article, ‘Trust and Antitrust’, Baier describes the relationship between asymmetric dependency and trust with an example of parents’ dependence on kindergarten [16]. By trusting the kindergarten staff to care for their children, parents also designate certain discretionary powers to the kindergarten and have to follow rules and procedures. Baier observes that when one person trusts another, one depends on the other’s good will and consequently, is also vulnerable to its limits. She explains, “*Exploitation and conspiracy, as much as justice and fellowship, thrive better in an atmosphere of trust*” ([16]: 232). Thus, trust alters relative power positions between the truster and the trusted with varying degrees of vulnerability.

Baier also refers to relations that change with social powers, especially with regard to social vulnerability. Conceptualizations of power within the theory of structuration, bio-power and reproductive autonomy from a feminist perspective significantly contribute to the discourses on social power. Weber’s idea of power as a capacity suggests that power is used as an instrument of domination, since there is an unequal relation between those who employ power and those who are subject to its effects [17]. Weber emphasizes the quantitative capacity of power that may be put to work, wherein the wishes of those with more power prevail over those with less power. Giddens’ conceptualization of power within the theory of structuration refers to a duality of structure; one structure is the capacity of one or more agents to enforce, and the other is a social community which reproduces the structure in which actions take place [18]. Foucault sees power everywhere, as described in his words: “*Power can retreat here, re-organise its forces, invest itself elsewhere and so the battle continues*” ([19]: 56). Foucault emphasizes that power produces effects at the level of desire and knowledge, suggesting that any action of power involves an increase in knowledge. The notion that power is only repressive, weakening and vacillating is inadequate and mistaken, according to Foucault.

Feminists have advocated for women’s reproductive right to enter into surrogacy contracts, based on their choice. Shalev (1989) asserts that surrogates should be allowed to disassociate from all parental rights and responsibilities prior to the birth of the babies, since women are rational beings in control of their emotions and also capable of making decisions about their bodies [20]. Laura Purdy (1996) argues that generalizing choices may be inadequate, especially in societies marked with limited choices [21]. This line of thought criticizes surrogacy as a practice subjugating vulnerable women and exploiting their reproductive capacity to serve the desires of wealthy couples. Through this lens, surrogacy is compared to organ transplantation, prostitution and slavery, and is seen as a paternalistic, medical and male-dominated establishment exploiting women. The Universal Declaration on Bioethics and Human Rights 2005 recognizes that technological advancements in medical science should be ethically sound, giving “*due respect to the dignity of the human person and universal respect for, and observance of, human rights and fundamental freedoms*” [22]. The Universal Declaration of Human Rights, Article 1 states, “*All human beings are born free and equal in dignity and rights. They are endowed with reason and conscience and should act towards one another in a spirit of brotherhood*” [23]. In other words, as Article 14 states, *“Everyone has the right to freedom of movement and residence within the borders of each state”.* Manifestations of exploitation will be examined from the above mentioned feminist and human rights perspectives.

**Figure 1:**
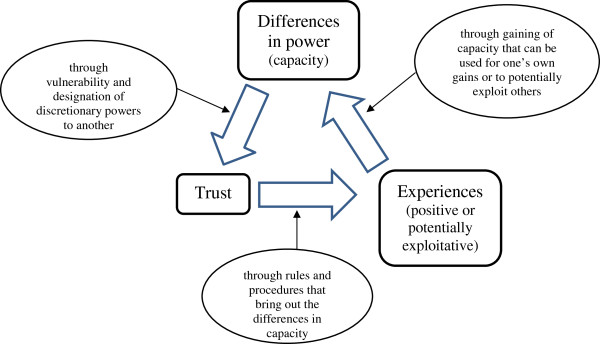
Framework of human relationships in surrogacy contracts.

Drawing on the above concepts, a framework is developed, linking trust, power and potential exploitation (Figure 1). In this framework, power is defined as capacity measured in terms of knowledge, contact and financial capability. Knowledge is characterized by understanding the medical process, legal issues, language and management of the surrogacy process. Financial capacity, in the context of surrogacy contracts, includes the intended parents’ (IPs) ability to pay for the required services, the SMs’ ability to withdraw from the contract or to file a legal case, and the medical practitioners’ (MPs) ability to provide the services with adequate medical equipment and personnel. Asymmetries of individual capacity (knowledge, contacts and financial capability) lead to trust amongst actors. This trusting process results in experiences that can either be positive or potentially exploitative. The experience can empower individuals with specific knowledge, contacts and further monetary gains. Drawing on Foucault’s conceptualization of power, this experience can lead to gaining knowledge and desires for one’s own benefit or for potentially exploiting other individuals and hence, a re-investment of power.

## Study area and methodology

This study applies approaches of ethnomethodology to identify phenomena as perceived by the actors in a situation, giving importance to their interpretations of the rules that make collective activity possible [24]. The methodology includes gathering information and perceptions using semi-structured interviews, discussions, participant observation, and explaining the phenomena from the perspectives of the research participants. A total of 13 SMs, 4 IPs and 2 doctors from one clinic in Western India were interviewed between August 2009 and April 2010. Ten SMs were first introduced through the clinic; later, three SMs were interviewed through the snowball method of referrals from initial respondents. The SMs were at different stages of the surrogacy process. Of the 13 SMs interviewed using the semi-structured questionnaire, 7 were in the process of surrogacy (SM1-7, one of whom was a second-time SM), 3 were tending the newborn babies post-delivery (SM10-13), and 3 had handed over the children and were at their respective homes (SM8-10). Ten of the mothers (SM1-7, 10–13) interviewed during the surrogacy process were interviewed again after they had completed the process. Of the 3 SMs caring for the babies post-delivery (SM10-13), one was at a child hospital with the newborn girl, waiting for the IPs to arrive from abroad. Another was staying at a hotel with the IPs, helping them tend the newborn babies, while the third SM was waiting at the surrogate home because the IPs wanted her to provide milk to the babies using breast pumps (SM13). Four IPs were interviewed using semi-structured interviews, 2 each from the continents of Europe and America.

The semi-structured interviews were recorded, translated into English and transcribed for reading and re-reading. In order to maintain confidentiality of participants, this article refers to SMs and IPs by numbers (SM1-13, IP1-4), does not identify the clinic’s name and location, and uses the IPs’ continents rather than countries of origin. The process of analysis included: transcription (not only of the literal statements recorded on tape, but also of the non-verbal gestures made during the conversations), bracketing and phenomenological reduction, listening to the tape and reading the transcription repeatedly to provide the context of smaller units of meaning for deriving emerging themes and delineating units of meaning relevant to the research question. These were then clustered into themes of emotions and experiences, such as knowledge, trust, coercion, fear, feelings of mistrust and dependency. This method of analysis has been used in previous public health qualitative studies [25,26]. The topics covered in the semi-structured interviews included socio-economic background, motivation and experiences of rules, living in the surrogate home, bonding, financial dealings, relinquishment and post-relinquishment. There were several opportunities for observing experiences through participation during the study, and these were noted in diary notes.

Written consent from the clinic’s principal MP was obtained by email before this researcher’s arrival in India. Written consent was also secured from the research participants, using consent forms. The consent form was translated into Hindi and Gujarati by local professional translators and was written in simple language. All SMs could read and hence gave their consent in writing. There is no formal process of ethical screening at the University of Heidelberg, Germany. However, a research team from South Asia Institute and Karl Jaspers Centre Heidelberg, as well as a few invited international research experts, generally discussed and screened the proposed study, both structurally and ethically, at the workshop, “Making India a Global Healthcare Destination: Historical and Anthropological Enquiries on Cross-border Healthcare”. This was co-organized by the Cluster of Excellence ‘Asia & Europe’, Heidelberg, and the French Institute of Pondicherry, it was held in Heidelberg from 14 to 15 June 2009 ^a^.

Drawing on the above-mentioned framework (Figure 1), this paper aims to examine the manifestations of exploitation during the commercial surrogacy process in the context of trust, power and experiences of actors, using a case study of one clinic in India. The findings of the study are hence structured into the following elements: capacities of actors, network of trust, requirements from actors, experiences of the surrogacy, manifestations of exploitation within the surrogacy process and re-organization of power.

## Characteristics and capacities of actors

The actors involved in the commercial surrogacy contract in India include the MPs, the IPs, the SMs and the surrogate agents (SAs). The MPs play a major role of managing the surrogacy process in India and in the study clinic. They have the medical knowledge and the in vitro fertilization (IVF) facilities (equipment and personnel) required for the process. They are familiar with the local procedures for providing birth certificates to the newborn babies. They use local contacts for providing the IPs with practical support, such as lodging, transportation, money exchange, neonatal care services and translators. This clinic owns two apartments for accommodating SMs during the surrogacy process.

They have contacts with SAs (mainly ex-surrogates or persons closely associated with the clinic) who introduce potential SMs to the clinic. The MPs formulate most of the regulations to be followed by the SMs, IPs and SAs. They do not allow homosexuals to enroll as IPs for the surrogacy process in this clinic, as the MPs believe in heteronormativity. However, they modify some regulations as required by the IPs, such as providing care for the children. They charge a fee of 1,100,000 rupees (approximately 15,700 euros) for one surrogacy process. One intended mother said the fee was doubled for twins (IP4). The MPs control the payment scheme (discussed in detail in the section on financial transactions). The MPs also use their knowledge and contacts in certain cases to evade the law. In one particular case, the twins born for one intended couple from Europe (IP3) were given false birth certificates. The children’s birth was registered with the SM’s name as ‘mother’ and the intended father’s name as ‘father’; the couple faced a legal struggle when caught, while the MPs were unaffected. A strong network is formed amongst IVF clinics operating in Western India that supports the doctors’ fraternity. The fraternity is influential not only in monitory terms, but also in contacts with the officials.

### Capacity of the intended parents

The IPs include couples from within India or abroad with a desire for a child. Faced with fertility issues, they have previously tried other options, such as IVF treatment and adoption, before opting for surrogacy, and have the financial capacity to initiate the process. One couple from America (IP1) had adopted one girl from another Asian country before coming to India for surrogacy. For this surrogacy process, the couple used the husband’s genetic material and their friend’s egg. The intended father was working in an information technology (IT) firm and the mother was a Human Resources Director in a mobile application firm. The second American couple (IP2) had their first daughter through a normal process; however, as they were unable to conceive again, they selected surrogacy. The intended father was an IT firm owner and the intended mother was a housewife. After an online research on several clinics in India, the intended mother chose this clinic, since it monitored the SMs throughout the pregnancy.

The factors that attract foreign couples to India for surrogacy include liberal laws, low cost, easy availability of women willing to become SMs, and their lesser rights in India. One intended mother (IP1) found the financial deal with this particular clinic convenient because it did not charge any upfront payment until the baby is handed over. In her words,

“*One of the things that made me come to this clinic was the way the payment scheme works. Only a nominal payment is made to the surrogate mother, but you don’t actually pay until the very end…it’s a good incentive for her (the SM) to keep the baby and not do much work so she doesn’t miscarry. She (the SM) doesn’t really get compensated until she hands over the baby*” (IP1).

The procedure in India was perceived as comparatively simple. One intended mother explained, “*Although it is legal in my country, the process is very complex and much more expensive than [in] India. The law expects surrogate mothers in India to sign over all rights to the baby even before the surrogacy begins, which is a big relief*” (IP1). The same intended mother also preferred this clinic because it did not require much background information about her. In her words, “*Another clinic I contacted expected me to fill up a huge form and then keep a time table and establish a protocol even before I came to India. But this doctor didn’t want to know anything about me until I arrived here, met her and trusted her*” (IP1). Both of the American intended mothers said they chose this particular clinic because the SMs were monitored at the surrogate homes. Hence, IPs chose one particular clinic based on its convenience, after reviewing several websites. The convenience in selecting this clinic included initialization procedures, payment scheme and management of SMs.

One intended mother (IP3) thought the law in India could be easily evaded. Surrogacy is banned in her country of origin. *“This is a poor country; everything can be done by paying money and people’s mouths can be shut”.* Similarly, IP4 thought they could manipulate surrogacy in India, although it was illegal in their country of origin. One MP was unhappy about countries that ban surrogacy as it leads to unpleasant legal struggles and hostile relationships with the IPs. Despite this, there were IPs from several countries that ban surrogacy who had already started the process in this clinic.

The IPs were at liberty to choose the kind of services they required after the child’s birth. Some parents preferred to hold on to the SM for a longer time to help them care for the baby. Others also wanted her to breastfeed to boost the child’s immunity. One intended mother who had two children said, “*The boy is healthy but the girl needs direct feeding, as her weight is not increasing, and I would prefer the surrogate mother to breastfeed her*” (IP2).

The IPs were unable to speak the local language and did not have any knowledge of the legal guidelines. They depended on the clinic for practical support, such as contact with hotels, cars for local transportation, foreign money transfer, a translator for communicating with the SMs and local people and neonatal care for pre-term babies. The clinic required all financial transactions between the IPs and the SMs to be made only through it. Hence, the IPs depended on the clinic to make the payments and trusted the doctors to transfer the money to the SMs. In one conversation with the intended mother, the doctor said, “*I will transfer the money to her (SM’s) account; you can trust me*”, to which the intended mother replied, “*I implicitly trust you*”. Hence, the IPs depended on the clinic for all sorts of support during the surrogacy procedure, and trust played a major role in the inter-relationship between the IPs and the MPs.

### Capacity of the surrogate mothers

All the SMs were facing household economic difficulties, so they chose to participate in surrogacy. Two women each had an ill family member; one had a child with severe disabilities; and another woman’s husband had a disease which needed immediate medical treatment. Three women found it difficult to make ends meet due to small earnings. One woman had a husband who suffered from addiction, and who spent most of his income on this habit. Four women wanted to save money for their children’s education. Two women wanted money to rebuild their *kachha* (unstable, temporarily built) house. One wanted to buy a house, since most of the family’s income was spent on rent. All the SMs could read and write, but none had studied beyond higher secondary level; 2 had completed up to 12^th^ grade, 6 up to the 10^th^ standard, and 5 had only completed primary education. According to their educational capacity, their family income was between 3000 and 6000 rupees per month (€50 to 100). Of the 13 SMs, 5 were domestic helpers, 3 were housewives, 1 was a clerk, 1 was a nurse, 2 were agricultural labourers and another worked in the family’s farm land. The remuneration they received through surrogacy is equivalent to 20 years’ worth of their salary; for them, it was a fortune. Their financial need was aptly expressed in the words of one surrogate mother:

*“This process is so distressing that I would not have done it even if someone paid me 10 times the remuneration, had I been well-off, but I am so desperate (for money) that I would do it even if I was paid just one third the amount”*.

According to the ART Bill, the surrogacy agreement is legally enforceable, but the drawback in this clinic was that SMs received no copy of their signed contract. This limits their capacity to file a lawsuit in case of breach of contract. They do not know the details of the ART Bill or their rights and duties under it, but they are aware that they are being exploited by not receiving a contract copy. Furthermore, given their socio-economic background, they may not even have the financial capacity to file a legal case. However, second-time SMs have an advantage over first- attempt SMs in this clinic. Based on their rapport with the clinic, SMs can repeat the surrogacy process with the same clinic and get paid a higher remuneration. To examine their familial situation post-surrogacy, all the SMs were visited again post-relinquishment at their homes. It was evident that they were all planning for another surrogacy. After further discussions, it became clear that some of the men had left their jobs and were coercing the women to participate in surrogacy again. Some feminists may be of the opinion that this is a matter of women’s reproductive autonomy and decision-making power. However, it needs to be seen how this familial equation changes further when women lose their reproductive capacity due to age or other medical factors.

### Capacity of the surrogate agents

Surrogate agents are ex-SMs or persons in close contact with the clinic, such as nurses, employees or other individuals connected with the clinic. Women who want to become SMs generally have to contact the clinic through an SA. Among ex-SMs, only those with a good reputation of having complied with all rules during their association with the clinic could become SAs. Through their experience, they know the surrogacy rules and procedures, as well as the means to evade some of these rules for their benefit. They have local contacts and have developed a rapport with the clinic to introduce potential surrogates. They are given a commission of 10,000 rupees on successful relinquishment of the baby. Hence, the clinic expects them to help with a smooth handover and counseling of the SM. Their commission is approximately equivalent to six months’ earnings.

Ex-SMs look for women in dire need of money to coerce them into surrogacy with the remuneration. As some ex-SMs participated in clinical drug trials ^b^, this was one of the openings they looked for in recruiting potential SMs. The first-time SMs knew nothing about the surrogacy process, so the SAs explained the basic scientific procedures involved, especially to convince the women that this process did not involve any physical intimacy. The SMs were informed about the clinic’s regulations and requirements. However, SAs were selective in giving information to SMs, such as failing to divulge the painful pre- and post-embryo transfer injections or the kind of medical interventions involved in the process. One SM already in the surrogacy process complained to an SA, “You never told me that this (the injections) is so painful”. The SA replied, “*Are you not getting all that money in return*?” Another SM admitted, “*I didn’t know these injections are so painful; now I feel like returning home immediately*”. The prospective SMs were also not informed that they may have to look after the child after birth. Agents also know the means of evading the clinic rules if required. This clinic has certain rules about the proof of residence of the SMs, but the SAs could manipulate this with false information.

### Asymmetries of capacity lead to a network of trust

**Figure 2:**
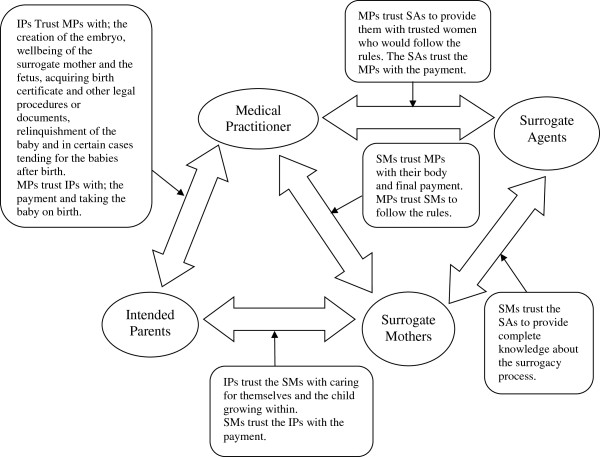
Asymmetries of capacity lead to a network of trust.

Surrogate mothers are most vulnerable due to their need for money and asymmetric capacity, compared to other actors. The IPs are also vulnerable due to their need for a child and lesser capacity, compared to the MPs. These asymmetries of capacity lead to a network of trust and designation of discretionary powers. In the context of commercial surrogacy in India, the IPs trust the MPs in creating the embryo, attending to the well-being of the SM and the fetus, and post-birth care giving. They may not have local contacts or knowledge of legal procedures (especially the IPs from abroad). Hence, they trust the MPs with these procedures. The SMs trust the MPs with their care and well-being during the surrogacy process, and the MPs trust the IPs with taking custody of the child and the payment at the end of the process. The MPs also trust the SMs to abide by the rules during the process. Hence, the asymmetries of capacity lead to a network of trust amongst actors (Figure 2).

## Requirements from actors

All the SMs undergo a medical screening to ascertain their reproductive capacity and general health status. According to the ART Bill, ”*No assisted reproductive technology procedure shall be performed on a woman below 21 years of age and any contravention of this stipulation shall amount to an offence punishable under this Act*” (5, Clause 20, 14: 16). However, one SM who participated in this study was under 20 years old. The ART Bill states, “*A surrogate mother shall not act as an oocyte donor for the couple or individual, as the case may be, seeking surrogacy*” (5, 34, 13: 27). Therefore, she has no biological claims over the child she carries. A woman who wants to participate as an SM is also required to obtain her husband’s permission by affixing his signature on the contract. One SA told me that this can be manipulated; women manage to bring any family member/friend posing to be her husband.

Article 9 of the Universal Declaration of Human Rights states, “*No one shall be subjected to arbitrary arrest, detention or exile”*[23]*.* However, it was mandatory in this clinic to confine all SMs in surrogate homes, away from their families, and impose house rules for the entire pregnancy period and for as long as required after delivery. The other requirements for SMs included; staying at the surrogate home for one year, following all the medical procedures, and caring for the baby after birth or as required by the IPs. The SMs were also expected to breastfeed the babies and look after them for as long as the IPs wanted.

The IPs were legally required to produce official documents that they can bring the children back to their homeland. However, this was not strictly followed; thus, some parents had to face legal hurdles. In July 2010, consulate generals of Belgium, France, Germany, Italy, the Netherlands, Poland, Spain and the Czech Republic sent letters to the most sought-after clinics in India, stressing the importance of redirecting nationals from their countries to their respective consulates before initiating the surrogacy process.

The SAs needed to have a close contact with the clinic and basic knowledge of the surrogacy process. The clinic required ex-surrogate SAs to have completed at least one surrogacy process and maintained an amicable relationship with the clinic. They were paid their commission only after successful relinquishment of the baby. While all the other actors followed the clinic’s rules, the MPs were answerable only to the court of law in India.

## Experiences of actors

The experiences of actors were examined at different stages of the surrogacy process: recruitment, medical procedures, living in the surrogate home, bonding with the child and amongst actors, financial dealings, relinquishment and post-relinquishment.

### Recruitment

Most SMs had some sort of financial problems that motivated them into surrogacy, as mentioned earlier (section on the capacity of the surrogate mothers); however, in the process, they also claimed it gave them satisfaction to be involved in an undertaking that is humane and brings happiness to other people’s lives. Apart from the previously mentioned selection criteria in recruiting potential SMs (section on the capacity of the surrogate mothers), other factors were reported by actors. Courteous and submissive conduct was an important criterion in selecting SMs. Women who showed signs of assertive behaviour were politely rejected by the clinic on medical grounds. The MPs noted that they expected their potential SMs to be healthy, clean, understanding and conscious of their responsibility. The long waiting list of women willing to participate in surrogacy made interchangeability possible. The IPs selected their SMs after face-to-face meetings at the clinic. For IPs who did not speak the local language, the clinic provided nurses as translators who could speak both languages. The IPs said they assessed their SM by her healthy appearance, willingness to relinquish the baby, family situation, husband’s occupation, medical history and family mortality history. The IPs also had religious preferences in selecting SMs. According to the MPs, 30% of couples insisted that the SM should be of the same religion as themselves, which manifests religious preference that can be seen as both/either personal bias or a potential discrimination. The Universal Declaration of Human Rights, Article 2, states, “*Everyone is entitled to all the rights and freedoms set forth in this Declaration, without distinction of any kind, such as race, colour, sex, language, religion, political or other opinion, national or social origin, property, birth or other status*” [23]. After this selection procedure, the SM signed a contract, followed by the embryo transfer process. In case the SM did not conceive after one round of embryo transfer, she would have to go through the same procedure with another couple, since this clinic did not allow SMs to repeat the process with the same couple.

### Medical procedures

The SMs mentioned some medical practices that they found unacceptable or undesirable. One SM had multiple embryos transferred at one time to her womb, and when more than one embryo were conceived, selective abortion was done. Due to the unnecessary medical intervention, the SM suffered a miscarriage of all the babies, and according to her, the attempt was wasted. The SMs also reported that invariably, all babies were delivered by caesarean section. One SM had started her labour pains, but was hurriedly operated on. One SM expressed the fear that if they rebelled against any clinic activity, the MPs were capable of performing an abortion and claiming that it happened naturally.

### Surrogate home

As mentioned earlier (section on requirements from actors), this clinic mandated that all SMs stay at the surrogate homes where certain rules had to be followed. The SMs were not allowed to do any house work and were expected to rest most of the time. Domestic helpers washed clothes, cooked food and cleaned the house for them. They had to eat only the food provided by the clinic. The clinic closely monitored their diet and activities at the surrogate home. They were restricted from eating spicy food; however, many women occasionally ate spicy food such as *samosas*^*c*^*,* since they were fed up with eating bland food every day. There were complaints about the inferior food quality. According to an SM, the matron pocketed part of the food budget. When SMs objected to the poor quality of fruits and food served, the matron said, *“This food is much better than what you eat at home… so eat it quietly”*.

After embryo transfer, SMs were expected to remain strictly in bed rest for 15 days. Following this, they were allowed to move around but had to stay on the first floor of the surrogate home for a three-month period. For instance, they were not even allowed to use the stairs to participate in baby shower ceremonies held on the ground floor, for fear that they might fall down and something might happen to the baby in the womb. According to the SMs, *“We have to be extra careful because this is not our child”.* Some SMs were happy to remain at the surrogate home as an escape from daily household chores, and for others, as a respite from domestic problems, such as drunken husbands. However, others were unhappy to leave breastfeeding children at home. One SM had to place her child with a disability at a special home. Children were allowed to visit their mothers only on Sundays, under several restrictive conditions. In case other SMs staying in the room complained, children were asked to leave the premises. One SM said her child had to be told that she had contracted a contagious disease or he would have insisted on living there with her. She also said that her child was not allowed to sit on her bed or hug her, lest this cause a miscarriage.

The SMs were generally bored for one year at the surrogate homes. Two surrogate homes did not have any television or radio. One surrogate home had television; however, disputes about its use arose among SMs. Some SMs who had been recommended by the matron had more control over entertainment media, such as television and radio. One quieter SM said she kept away from such troubles. Overcrowding resulted in water and hygiene issues that caused concern amongst the SMs and conflicts within these homes. During special occasions, such as baby showers or visits from a media crew, doctors or IPs, the matron made arrangements to clean up the surrogate home thoroughly and provided good food to the SMs. One SM’s spouse commented that it is the IPs’ duty to take more interest in the SMs’ condition during the pregnancy and their provisions within the surrogate homes. The SMs complained of water shortage, cramped conditions, substandard food quality, and poor sanitation and hygiene at the surrogate homes. On completion of the 7^th^ month of pregnancy, all SMs were transferred to the surrogate home above the IVF clinic so that they could be monitored more closely.

### Relinquishment and bonding

The SMs in this clinic were expected to take care of the child after birth. The MPs trusted the SMs as the best caretakers of the babies, and they were paid a bonus for their services. Three post-delivery situations were observed; one was when the IPs arrived late and the clinic gave the SM the responsibility to care for the baby in a children’s hospital. The second situation was that the IPs arrived, but wanted the SMs to provide their breast milk. In the third situation, the IPs expected the SMs to move into their hotel as full-time nannies for the babies. The arrangement for the children’s passports took approximately eight weeks; during this period, the SMs remained with the IPs, taking care of the babies. Some IPs expected the SMs to breastfeed the babies, while others did not want direct breastfeeding, so SMs were asked to stay at the surrogate home above the clinic to supply the milk using breast pumps. The SMs themselves were happy to do this for the babies. According to them, “*This was the only opportunity for us to interact with the children. We cherished these days; as we all know, once these children are gone, they will never come back*”. They took the babies’ pictures during this time and kept these. All the SMs showed these baby photos to this researcher while sharing their experiences. They sympathized with women who were unable or restricted from this interaction with the babies.

The clinic decided and organized the process of relinquishment to suit the IPs’ convenience. The SM was expected to be a rational person in control of her body and emotions at the time of relinquishment. One SM waited for the IPs’ arrival from Europe for three weeks. She and her husband looked after the baby girl during this period, breastfeeding, changing nappies and providing all other required care for the child. Their attachment to the baby was evident in their affectionate kissing and fondling, either in response to distressed cries or during play time. The couple arrived from Europe 21 days later and immediately took the baby with them. They were shifted to the surrogate home above the clinic, awaiting their payment. The IPs came to the clinic two days later, paid the doctor for all the services and left. They did not attempt to meet the SM, although she was upstairs in the same building. Most of the IPs did not want to keep any contact with the SMs after the process. Moreover, very little social and psychological support was given to the SMs in the clinic, leaving them feeling miserable post-relinquishment. One SM shared her experience of being scolded by a nurse: “*Why are you crying now; didn’t you know this is what would happen when you started this process*”.

One SM was hopeful that the IPs would keep in touch with her after leaving. She had told them, “*I don’t want anything else, but please send the photographs of the children at least once a year*”, and the IPs had promised to do so. A few months later, she was still waiting for their phone call. She confided to me, “*My falling hair began recently; I think the children have started smiling. In our culture, we believe that when our babies start smiling and laughing, the mother loses her hair*” (SM13). Eight months later, when she did not receive any phone call or mail from them, she was very unhappy with the couple and considered their behaviour utterly selfish. Other ex-SMs also described similar experiences of relinquishment and feeling empty after the couples’ departure with the babies. One SM was given wrong contact details by the IPs.

Based on her surrogacy experience, IP1 observed that the clinics have a responsibility to prepare the surrogate and parents for the child’s arrival and the separation, which is not done to the level that it should be.

“*The clinics should counsel parents and surrogates and have to determine what kind of relationship each individual case is going to have. They can’t prescribe the same set of rules to each surrogate and parent and patient group. This reminds me of adoption in my country and how it is either ‘open’ or ‘closed’ with respect to the relationship with the birth mother. Some want the openness and some don’t; it’s very individual. Clinic staff can help facilitate respectful relationships” (IP1).*

According to the doctor, the triangle of emotions amongst the SM, the child and the couple is:

“*Nothing but a false idea. The surrogate mother is prepared right from the beginning and taught that the child is not hers, [whom] she has to give over to the couple, as [the child] rightfully belongs to them. As a result, right from the beginning, the feeling of the surrogate mother towards the child is trivial*”.

According to one IP, *“From the start, the doctors try to counsel the surrogate, making her aware that the baby was not hers to ‘give away’, but results from the embryo belonging to the biological parents* (or donors)”.

Regarding psychological counseling, the doctor said,

“*No feelings ever develop amongst surrogates for the child, so the question of resolving any feelings does not arise*. *The place of feelings for the child is taken over by the money in the case of the surrogate mother. In the entire process, she gives more importance to the monetary factor, while the feelings for the child are less. There might be some feelings towards the end, but as the memory blurs over a period of time, these too fade away. Finally, her feelings for the child become non-existent”.*

Regarding the child’s feelings, the doctor’s opinion was:

“*Do you remember what or whom you saw when you [first] opened your eyes in this world? If you answer truthfully, it will of course be ‘no’*. *So no feeling ever develops in the child for the surrogate mother”*.

Two of the four IPs in this case study had kept in contact with the SMs. They recognized and accepted the bond between the SMs and the babies, and intend to tell their children about the circumstances of their birth. These parents took the SMs along with them to the hotels, booking separate rooms for them and the children. The SMs cared for the babies while the IPs themselves took time to bond with the babies. One IP initially felt detached from her children at that time; she said, “*The children don’t respond to my voice; however, when she* (the SM) *speaks, they immediately respond to her voice and tone*”. IP1 was here alone without her husband, waiting for her children’s passports from the embassy. She was not involved in any of the children’s activities, such as bathing, feeding or tending her babies. In contrast, IP2 felt intimate with her children soon after birth and was involved in bathing and caring for them (one girl and one boy).

In requesting the SMs to breastfeed their babies, the IPs were concerned about the health and well-being of the children. According to one intended mother, “*She* (the baby girl) *was very small so she still needed some breast milk; she got formula too. This guy* (the baby boy*), I gave him breast milk just once to keep his immune system running*” (IP2). She also immensely trusted the SM with her babies, *“I treat my surrogate like my sister. I can at any time leave my babies with her and go. I have a nanny to help her in the room. The nanny does all the work”*.

IP1 was in touch with the SM through phone calls. She sent gifts when her friend visited India. When this researcher visited the SM, the intended mother sent the children’s photos and messages for the surrogate mother to this researcher by email. The intended mother had not given the SM her contact information, so she could contact the SM, but not vice versa. Similarly, this researcher was also a contact person if the SM wanted to reach the intended mother. According to the SMs, most IPs do not want to maintain any relationship with them. The doctors also convinced the IPs that the SM had been paid for all her services, and there was no need to feel indebted or contact her further, since she did not want that. According to the doctor, “*The surrogate mother herself does not wish it to be so (to keep contact with IPs and the child). The money she receives in return plays an important part in this*”. However, the SMs said they wanted the IPs to keep in touch and most have tried to speak about this to the IPs, but have been unable to convey their message due to language barriers. The SMs felt the doctors and IPs cared for them only until they handed over the baby.

### Financial transactions

The MPs controlled the payment scheme within the surrogacy process. The SMs needed money due to some financial difficulties or insecurities, but they were not paid until they had handed over the baby and completed all the requirements of the surrogacy process. The IPs needed a child; although the child was handed over, official documents such as birth certificates were issued only after most of the surrogacy payment had been made. The MPs made these decisions. All the payments made by the IPs to the SMs had to be channeled through the clinic. The SMs were paid a monthly amount of 2,500 rupees (Table 1). The first lump sum payment of 25,000 rupees was made after completion of 4 months of pregnancy and again after 8 months of pregnancy. The main payment of 177,500 rupees was given to the SM after she handed over the baby. If there was miscarriage at any stage or stillbirth, SMs were not paid any extra amount. The MPs requested the IPs for an additional payment to be given to the SMs for breastfeeding and neonatal care services, or merely as a bonus. This sum was not fixed and depended on the IPs’ financial capacity. The SA was paid 10,000 rupees on successful relinquishment.

**Table 1:** Payment installments made to the surrogate mothers and by the intended parents

**Payment to the surrogate mother**	**In rupees**	**In euros**
Monthly payment (for her household expenditure as she stayed in the surrogate home).	2,500	36
On completion of 4th month	25,000	357
On completion of 8th month	25,000	357
On handing over the baby (Rs 10,000 to be paid to the surrogate agent).	177,500	2,535
Total amount paid to the surrogate mother	250,000	3,430
**Payment by the intended parents to the clinic (this rate was doubled in case of twins).**	1,100,000	15,714
Approximate additional costs incurred by intended parents (including transport cost, caesarean section, breastfeeding, neonatal clinic and official documents).	1,400,000	20,000

The SMs were generally not satisfied with the remuneration. According to the doctor, “*Eighty percent of the surrogate mothers are not happy with the money they receive. Compared to her work* (as a surrogate mother), *this sum is relatively less, but the economic background from which she comes is such that for her it appears to be the biggest gift in life*”.

The couples spent approximately 20,000 euros (1,200,000 rupees) for the surrogacy process, which one intended mother (IP4) reported was doubled in case of twins. However, the payment for the SMs remained the same, except some money paid as a bonus. The official amount paid to the SMs was 250,000 rupees (€3,430); however, this varied from case to case as they were paid extra charges for breastfeeding, tending the baby and some bonus for additional children. The IPs made this extra payment based on their capacity. In this case study, the highest payment made to one SM was 500,000 rupees (€7,143). This particular intended mother wanted to pay more, but the doctor deterred her, saying, “*This could prompt such demands and unnecessarily raise expectations from other surrogate mothers as well*”.

## Manifestations of exploitation

Surrogate mothers were confined to surrogate homes, not given a contract copy, subjected to unnecessary medical interventions, not provided with medical insurance, and expected to breastfeed and bond with the children without any psychological counseling. These are all manifestations of exploitation and violation of basic human rights, as stated in Articles 1, 2, 9 and 14 of the Universal Declaration of Human Rights and The Universal Declaration on Bioethics and Human Rights 2005 [22,23]. The SMs’ dissatisfaction with the remuneration, some medical practices, and the manner in which their relationship with the IPs is managed, indicates that given more decision-making power, they would express their preferences in the process. The SMs were paid for handing over the children, not for their reproductive capacity, as they were not compensated at all in case of miscarriage at any stage of the pregnancy or during the birth process. The SMs’ financial motivation and lack of rights and decision-making power reveal that feminists’ concerns regarding subjugation of vulnerable women and exploitation of their reproductive capacity, especially in societies marked with limited choices, are relevant in this case study in India.

Although the MPs were in an interrelation of trust with other actors (IPs, SMs and SAs), they had maintained control over the actors because of their capacity. They are in command of the payment scheme, the medical interventions, interaction between the SMs and IPs and the relinquishment. The IPs can choose the clinics and SMs based on their preferences; hence, the MPs are pressured to provide specific services to attract IPs as clients. The IPs are also exploited on fees, as they are not provided with complete information about possible extra costs they would incur after delivery. There are additional expenses to be paid to the SMs, to the neonatal hospital (most children are pre-term), and for official documents and the caesarean section.

## Conclusion

This study reveals that asymmetries of capacity amongst the MPs, SMs, IPs and SAs lead to a network of trust and designation of powers through rules and procedures. These circumstances bring out the relevance of Baier’s conceptualization of asymmetric vulnerability, trust and potential exploitation in human relationships. The IPs are exploited, especially in monetary terms. The SMs are relatively the most exploited among the actors, given their vulnerability. The remuneration they receive through surrogacy is significant, and the knowledge they gain as ex-surrogates is used for their own benefit and for exploiting others. Foucault’s conceptualization of power is thus relevant in the case of ex-SMs, since their exploitative experience results in a re-investment of power.

The implications for ethical debate include the questionable conditions in which choices are made within the surrogacy process, the role of the medical system and the question of the IPs’ social responsibility [27]. Ethical arguments to justify commercial surrogacy, based on the assumption of the rational choice of entering into contracts and mutual benefit, are ignorant of social and cognitive conditions in a structurally unjust system. A critical assessment of the role of doctors and the medical system (their gatekeeper function is highly questionable) reflects the objectification of SMs in the surrogacy process. At present, the MPs play a major role as agents of the surrogacy process and consequently, have an upper hand in decision making and stipulated rules. The clinic’s function needs to be isolated from its role as a surrogacy agent to reduce vested interest and authority of a single institute in the process, especially since it is a profit-making institution exploiting the vulnerability of both SMs and IPs. Finally, questions of social responsibility are also directed towards Western IPs when public knowledge of exploitation of SMs increases.

## Endnotes

^a^This study was conducted by Sheela Saravanan as a Post Doctoral Researcher at the Cluster of Excellence, Asia and Europe in a Global Context, University of Heidelberg between July 2009 to June 2010. http://www.ifpindia.org/ecrire/upload/meetings/Workshop_Heidelberg_June_2009.pdf.

^b^The number of approved Global Clinical Trials (GCTs) in India rose sharply from 65 in 2008 to 391 in 2009, which continued to rise with 500 GCTs being allowed in 2010, 325 in 2011 and 262 in 2012. Recently the revelation that 2,262 people had died in these trials during the past five years led to a public outcry and the Supreme Court intervened with stricter norms for controlling drug trials. Indians were being used as “guinea pigs” in these drug trials and the Supreme Court had criticized the Health Ministry for allowing this [28].

^c^A samosa, a popular South Asian snack, is generally a fried or baked triangular pastry stuffed with spiced potatoes.

## Competing interest

I declare that I have no significant competing financial, professional or personal interests that might have influenced the performance or presentation of the work described in this manuscript.
